# An Analysis of Trainee Salaries Offered in Nurse Practitioner and Physician Assistant/Associate Postgraduate Residency and Fellowship Programs in the United States

**DOI:** 10.7759/cureus.64434

**Published:** 2024-07-12

**Authors:** Vasco Deon Kidd, Jessica L Horstmann, Shayanna Felder, Kerry Bamrick

**Affiliations:** 1 Orthopedic Surgery, University of California Irvine School of Medicine, Irvine, USA; 2 Community Health, Native American Community Clinic, Minneapolis, USA; 3 Accreditation, Consortium for Advanced Practice Providers, Newport Beach, USA

**Keywords:** postgraduate np pa training, trainee stipend, trainee salaries, nurse practitioner, advanced practice registered nurse, physician associate, physician assistant, advanced practice providers, residency and internship, fellowship training

## Abstract

Background

Although there has been steady growth in the number of postgraduate nurse practitioner (NP) and physician assistant/associate (PA) residency and fellowship programs in the United States, little is known about annual salaries paid to trainees across a national sample of postgraduate programs and specialties. We describe postgraduate program NP and PA trainee salaries and the relationship to specific variables.

Methodology

An electronic survey was distributed via email to 336 postgraduate NP, PA, and joint NP/PA residency/fellowship programs between November 2023 and December 2023. Frequency tables (for categorical variables) and descriptive statistics (for continuous variables) were used to summarize the data. Chi-square tests of independence were used to determine the relationship between trainee salary and program type, geographical location, and clinical setting.

Results

There was a statistically significant association between trainee salary for primary care and clinical profession (χ^2^(6) = 13.993, p = 0.022). Over half of NP respondents (52.1%) reported that their trainees had an annual salary between $76000 and $86000. The majority of PA respondents (57.1%) reported that their trainees had an annual salary below $75000. Respondents who were non-clinical professionals (50.0%) reported that their trainees had an annual salary of over $86000. The single physician respondent also reported that their trainees' had an annual salary of over $86000. It appears that PA respondents were more likely to report lower trainee salaries than respondents who were NPs and non-clinical professionals. Additionally, respondents associated with primary care joint NP/PA cohorts were more likely to report higher trainee salaries than participants having NP-only cohorts. Lastly, there was a statistically significantly positive relationship between trainee salary and the number of postgraduate advanced practice provider (APP) trainees in psychiatric mental health (τ_b _= 0.451, p = 0.006).

Conclusion

To the best of our knowledge, this national study is the first of its kind to examine and summarize APP postgraduate trainee annual salaries across multiple specialties. Additional studies are needed to clarify the relationships between trainee salaries and other variables.

## Introduction

Advanced practice provider (APP) postgraduate training programs, also known as fellowships and residencies, encompass nurse practitioners (NPs) and physician assistants/associates (PAs). Typically spanning 12 to 24 months in duration, these programs differ from traditional entry-level APP roles in that trainees complete a formalized training program comprised of didactic sessions, quality improvement initiatives, specialty rotations, and scholarly projects [1,2]. Postgraduate programs are either single-track or multi-specialty track and are structured as NP, PA, or joint NP/PA programs [3]. Moreover, while multiple optional accrediting pathways exist for postgraduate programs, the majority remain unaccredited [3,4]. Although the benefits of employing NPs and PAs in healthcare are well elucidated in the literature, there remains insufficient data regarding APP postgraduate training impacts on patient outcomes and efficiency of care [5-10].

The primary financing mechanism for APP postgraduate training programs is internal institutional revenue or, in some cases, a highly competitive, time-limited grant [10,11]. In addition, annual APP trainee salaries vary across postgraduate programs and there is no consistent guidance or publicly available data to help program directors navigate this complex landscape independently. The question of fair and competitive annual salary poses a significant challenge for postgraduate training programs which must balance competitive recruitment initiatives with improving program solvency and return on investment.

There are numerous factors that may impact postgraduate APP trainee salaries such as geography, as market rates vary by geographical cost of living index by state. Other factors include program composition, specialty or subspecialty focus, and clinical settings (academic medical centers, federally qualified health centers (FQHCs), hospital/multi-hospital settings, the Veteran’s Affairs (VA) system, etc.). An evaluation of these factors related to APP trainee salaries has not been previously described in the literature.

In view of the multiple factors impacting annual trainee salaries, there is a need for foundational data to address research gaps and help inform decision-making on promulgating fair and equitable trainee salaries. The aims of our research were to summarize APP trainee salary data by program type, geographical location, clinical setting, and specialty track, and to explore any associations between annual trainee salary data and these four variables.

## Materials and methods

An electronic survey consisting of 27 items was developed by the research team. Before dissemination, the anonymous survey was piloted on a sample of postgraduate program attendees in July 2023 at the annual Consortium for Advanced Practice Providers conference in Washington, DC. Based on the feedback of the pilot group, the survey was finalized in October 2023. An email invitation with a link to a voluntary, anonymous, and online survey (Qualtrics Inc., Provo, UT, USA) was distributed to 336 active postgraduate programs. Specifically, we targeted program directors for the survey. For the purposes of this study, each individual specialty training track was considered a program, even if the training track was embedded within a larger program [3]. The email introduction to the survey contained all the necessary elements of written consent, and submission of the survey indicated the respondents’ consent to participate. Confidentiality was maintained throughout the study. The study period was from November 20, 2023, through December 31, 2023. Multiple email reminders were sent out during the study period to improve participant engagement. This study received approval from the Institutional Review Board (IRB) of Community Health Center, Inc. (approval number: 1207).

Analysis methods

Data were imported into and analyzed using Statistical Package for the Social Sciences (IBM SPSS Statistics for Windows, IBM Corp., Version 23.0, Armonk, NY). Frequency tables (for categorical variables) and descriptive statistics (for continuous variables) were used to summarize the data. Chi-square tests of independence were used to determine the relationship between trainee salary and program type, geographical location, and clinical setting. The chi-square test of independence is a statistical hypothesis test used to determine whether two categorical variables are related [12]. When the expected value of a cell in the contingency table was less than 5, the p-value of the chi-square test was computed by the Monte Carlo method [13,14]. Kendall’s tau-b correlation coefficients were used to determine if there was an association between trainee salary and the number of postgraduate APP trainees [15]. Kendall's tau-b is a nonparametric measure of the strength and direction of association that exists between two variables measured on an ordinal scale [16]. The analysis for the association between trainee salary and other variables was only performed for two specialties (Primary Care and Psychiatric Mental Health) after taking the restriction of sample size into consideration. According to Bujang and Baharum (2016), a sample size of at least 19 was needed to be able to detect a correlation coefficient of 0.6 (a medium effect size based on Cohen (1992) with an alpha of 0.05 and a power of 80%) [17,18]. For any tests, a p-value less than 0.05 indicated statistical significance.

## Results

The overall response rate was 45% (151/336). Two respondents were excluded from data analysis because they skipped the majority of the survey questions. Table 1 shows the demographics of the respondents. Approximately two-thirds of the respondents (64.9%) were NPs. The majority of respondents were located in New York 10.5% (16/151), California 7.2% (11/151), and Washington 7.2% (11/151). When grouping the states into regions, about one-third of respondents (31.8%) were from the Northeast region. Academic Medical Center (34.4%), FQHC (27.8%), and U.S. Department of VA (17.3%) were the primary sponsoring organizations of the APP postgraduate training programs. Respondents indicated that nearly half of the postgraduate APP trainee cohorts (47.7%) were NPs.

**Table 1:** Demographics The data has been represented as sample size (N) and percentage (%). APP: advanced practice provider

Participant Demographics	N (%)
Clinical profession	
Nurse Practitioner (NP)	98 (64.9)
Physician Assistant/Associate (PA)	41 (27.2)
Physician	5 (3.3)
N/A: Non-clinical	7 (4.6)
Region	
Midwest	33 (21.9)
West	39 (25.8)
Southeast	17 (11.3)
Southwest	14 (9.3)
Northeast	48 (31.8)
Sponsoring organization of the APP postgraduate training program	
Academic Medical Center	52 (34.4)
Federally Qualified Health Center (FQHC)	42 (27.8)
U.S. Department of Veterans Affairs	26 (17.2)
FQHC Look-a-like	2 (1.3)
Hospital	9 (6.0)
Multi-Hospital System	9 (6.0)
Private Practice	4 (2.6)
Other	7 (4.6)
Composition of the postgraduate APP trainee cohort	
NP only	72 (47.7)
PA only	19 (12.6)
Joint NP/PA	60 (39.7)

Regarding the number of postgraduate trainees, 145 respondents disclosed information for 43 out of 59 specialty tracks. The average number of postgraduate APP trainees ranged from 1.00 to 6.00 (Table 2). It should be noted that specialty tracks with only one respondent were not represented in Table 2 to improve the visual representation of the data by specialty track.

**Table 2:** Descriptive statistics of the number of postgraduate APP trainees per specialty track The data has been represented as N: sample size, M: mean, SD: standard deviation, min: minimum, max: maximum, NA: not available. APP: advanced practice provider

Specialty	N	M	SD	Min	Max
Acute Care	10	4.50	2.84	1	10
Adult-Gerontology/Internal Medicine	7	4.43	3.51	1	11
Cardiology	4	1.50	0.58	1	2
Cardiothoracic Surgery	5	1.60	0.89	1	3
Critical Care (Adult, Pediatrics, Surgery)	12	2.50	1.24	1	5
Emergency Medicine	14	3.64	3.34	1	11
General Surgery	6	4.67	3.56	2	11
Geriatric Medicine	4	5.00	4.08	2	11
Hematology and Oncology	3	1.67	0.58	1	2
Hospital Medicine/Hospitalist	6	3.33	2.34	1	6
Neonatal	2	2.50	0.71	2	3
Neonatal Critical Care	3	1.67	0.58	1	2
Neonatal Intensive Care	4	1.74	0.96	1	3
Neurology	8	1.38	0.52	1	2
Neurosurgery	3	1.00	0	NA	NA
Oncology	4	4.25	4.57	1	11
Organ Transplant	3	1.00	0	NA	NA
Orthopedics	5	1.60	1.34	1	4
Palliative	4	1.25	0.50	1	2
Pediatrics	6	2.50	1.76	1	6
Pediatrics Acute Care	3	5.00	3.61	1	8
Primary Care	62	4.40	2.64	1	11
Psychiatric Mental Health	30	2.70	2.07	1	11
Rural Family Medicine	6	5.17	2.79	1	8
Substance Abuse	2	4.00	2.83	2	6
Trauma Surgery	2	2.00	0	NA	NA
Transplant Surgery	2	1.00	0	NA	NA
Urgent Care	4	4.25	1.71	2	6
Urology	4	1.25	0.50	1	2
Women's Health	2	3.50	3.54	1	6

In terms of reporting postgraduate APP trainee annual salaries, respondents of the Southwest region (21.4%) seemed to be less likely to disclose trainee annual salary information than others (5.9-18.2%). In addition, respondents with the joint NP/PA cohorts (21.7%) seemed also less likely to disclose trainee salary information than others (9.7-10.5%). Table 3 provides a breakdown of respondents who either disclosed or did not disclose annual trainee salary data.

**Table 3:** Report of trainee salaries The data has been represented as sample (N) and percentage (%). APP: advanced practice provider

Characteristics	Information Revealed	
	Yes (N = 129)	No (N = 22)
Clinical profession		
Nurse Practitioner (NP), N = 98	85 (86.7)	13 (13.3)
Physician Assistant/Associate (PA), N = 41	36 (87.8)	5 (12.2)
Physician, N = 5	2 (40.0)	3 (60.0)
N/A: Non-clinical, N = 7	6 (85.7)	1 (14.3)
Region		
Midwest, N = 33	27 (81.8)	6 (18.2)
West, N = 39	35 (89.7)	4 (10.3)
Southeast, N = 17	16 (94.1)	1 (5.9)
Southwest, N = 14	11 (78.6)	3 (21.4)
Northeast, N = 48	40 (83.3)	8 (16.7)
Sponsoring organization of the APP postgraduate training program		
Academic Medical Center, N = 52	42 (80.8)	10 (19.2)
Federally Qualified Health Center (FQHC), N = 42	37 (88.1)	5 (11.9)
U.S. Department of Veterans Affairs, N = 26	24 (92.3)	2 (7.7)
FQHC Look-a-like, N = 2	2 (100)	0
Hospital, N = 9	9 (100)	0
Multi-Hospital System, N = 9	7 (77.8)	2 (22.2)
Private Practice, N = 4	3 (75.0)	1 (25.0)
Other, N = 7	5 (71.4)	2 (28.6)
Composition of the postgraduate APP trainee cohort		
NP only, N = 72	65 (90.3)	7 (9.7)
PA only, N = 19	17 (89.5)	2 (10.5)
Joint NP/PA, N = 60	47 (78.3)	13 (21.7)

Of the 59 specialties, 22 specialties had no data for trainee salary. For the 37 specialty tracks with the data for trainee salary, the salary information was summarized into seven categories (below $65000, $65000-$75000, $76000-$85000, $86000-$95000, $96000-$105000, $106000-$125000, and over $126000) as outlined in Table 4. The number of respondents that revealed the information for trainee salary ranged from 1 to 62 per specialty track. In regard to primary care (the specialty track with the most respondents), nearly half of the trainees (43.5%) had salaries within the range of $76000 and $85000.

**Table 4:** Cross-tabulation of trainee salary by specialty track The data has been represented as sample (N) and percentage (%).

Specialty	N	Below $65000	$65000-$75000	$76000-$85000	$86000-$95000	$96000-$105000	$106000-$125000	Over $126000
Acute Care	9	1 (11.1)	1 (11.1)	4 (44.4)	3 (33.3)	0	0	0
Administration Specialty	1	0	0	0	0	0	0	1 (100)
Adult-Gerontology/Internal Medicine	7	0	0	3 (42.9)	3 (42.9)	0	1 (14.3)	0
Cardiology	4	1 (25.0)	1 (25.0)	2 (50.0)	0	0	0	0
Cardiothoracic Surgery	3	0	1 (33.3)	2 (66.7)	0	0	0	0
Cardiothoracic Transplant	1	0	1 (100)	0	0	0	0	0
Critical Care (Adult, Pediatrics, Surgery)	10	2 (20.0)	2 (20.0)	3 (30.0)	2 (20.0)	1 (10.0)	0	0
Emergency Medicine	12	5 (41.7)	4 (33.3)	1 (8.3)	1 (8.3)	0	1 (8.3)	0
General Surgery	5	3 (60.0)	2 (40.0)	0	0	0	0	0
Geriatric Medicine	4	0	0	1 (25.0)	3 (75.0)	0	0	0
Hematology and Oncology	3	0	1 (33.3)	2 (66.7)	0	0	0	0
Hospital Medicine/Hospitalist	4	0	4 (100)	0	0	0	0	0
LGBTQ+ Health	1	0	0	1 (100)	0	0	0	0
Neonatal Critical Care	2	0	0	0	0	0	2 (100)	0
Neonatal Intensive Care	2	0	1 (50.0)	1 (50.0)	0	0	0	0
Nephrology	1	0	1 (100)	0	0	0	0	0
Neurology	5	1 (20.0)	2 (40.0)	2 (40.0)	0	0	0	0
Neurosurgery	1	0	1 (100)	0	0	0	0	0
Obstetrics and Gynaecology (OBGYN)	1	0	1 (100)	0	0	0	0	0
Oncology	2	0	0	1 (50.0)	0	1 (50.0)	0	0
Organ Transplant	2	2 (100)	0	0	0	0	0	0
Orthopedics	3	0	3 (100)	0	0	0	0	0
Palliative	2	0	2 (100)	0	0	0	0	0
Pediatric Comprehensive Cardiac Care	1	0	0	1 (100)	0	0	0	0
Pediatric Emergency Medicine	1	0	0	1 (100)	0	0	0	0
Pediatric Urgent Care	1	0	0	0	1 (100)	0	0	0
Pediatrics	6	1 (16.7)	1 (16.7)	1 (16.7)	2 (33.3)	1 (16.7)	0	0
Pediatrics Acute Care	3	0	0	0	1 (33.3)	2 (66.7)	0	0
Primary Care	62	0	10 (16.1)	27 (43.5)	18 (29.0)	5 (8.1)	1 (1.6)	1 (1.6)
Psychiatric Mental Health	26	1 (3.8)	4 (15.4)	11 (42.3)	5 (19.2)	3 (11.5)	2 (7.7)	0
Rural Family Medicine	5	0	1 (20.0)	2 (40.0)	1 (20.0)	1 (20.0)	0	0
Substance Abuse	2	0	0	0	1 (50.0)	1 (50.0)	0	0
Trauma Surgery	2	1 (50.0)	1 (50.0)	0	0	0	0	0
Transplant Surgery	2	1 (50.0)	1 (50.0)	0	0	0	0	0
Urgent Care	4	1 (25.0)	1 (25.0)	0	1 (25.0)	1 (25.0)	0	0
Urology	4	0	1 (25.0)	1 (25.0)	0	0	2 (50.0)	0
Women's Health	2	0	0	0	1 (50.0)	1 (50.0)	0	0

Table 5 shows the cross-tabulation of trainee salary for Primary Care and demographics and the results of chi-square tests. There was a statistically significant association between trainee salary for Primary Care and clinical profession (χ2(6) = 13.993, p = 0.022). Moreover, there was a statistically significant association between trainee salary for Primary Care and postgraduate APP trainee cohort (χ2(2) = 7.802, p = 0.016). However, there was no statistically significant association between trainee salary for Primary Care and region (χ2(8) = 4.248, p = 0.859) and sponsoring organization of the APP postgraduate training program (χ2(8) = 11.655, p = 0.139).

**Table 5:** Trainee salary for Primary Care by demographics The data has been represented as N: sample size, (%) percentage, Df: degrees of freedom, χ2: chi-squared, P: p-value. * indicates statistical significance at the 0.05 level. APP: advanced practice provider

Category	Primary Care (N = 62)					
	Below $75000	$76000-$85000	Over $86000	χ^2^	df	p
Clinical profession						
Nurse Practitioner (NP), N = 48	5 (10.4)	25 (52.1)	18 (37.5)	13.993	6	0.022*
Physician Assistant/Associate (PA), N = 7	4 (57.1)	0	3 (42.9)	-	-	-
Physician, N = 1	0	0	1 (100)	-	-	-
N/A: Non-clinical, N = 6	1 (16.7)	2 (33.3)	3 (50.0)	-	-	-
Region						
Midwest, N = 13	3 (23.1)	7 (53.8)	3 (23.1)	4.248	8	0.859
West, N = 22	2 (9.1)	8 (36.4)	12 (54.5)	-	-	-
Southeast, N = 5	1 (20.0)	2 (40.0)	2 (40.0)	-	-	-
Southwest, N = 4	1 (25.0)	2 (50.0)	1 (25.0)	-	-	-
Northeast, N = 18	3 (16.7)	8 (44.4)	7 (38.9)	-	-	-
Sponsoring organization of the APP postgraduate training program						
Academic Medical Center, N = 4	1 (25.0)	2 (50.0)	1 (25.0)	11.655	8	0.139
Federally Qualified Health Center (FQHC), N = 36	7 (19.4)	13 (36.1)	16 (44.4)	-	-	-
U.S. Department of Veterans Affairs, N = 13	1 (7.7)	10 (76.9)	2 (15.4)	-	-	-
Hospital/Multi-Hospital System, N = 4	1 (25.0)	0	3 (75.0)	-	-	-
Other, N = 5	0	2 (40.0)	3 (60.0)	-	-	-
Composition of the postgraduate APP trainee cohort						
NP only, N = 44	5 (11.4)	24 (54.5)	15 (34.1)	7.802	2	0.016*
PA only	0	0	0	-	-	-
Joint NP/PA, N = 18	5 (27.8)	3 (16.7)	10 (55.6)	-	-	-

Table 6 shows the cross-tabulation of trainee salary for Psychiatric Mental Health and demographics and the results of chi-square tests. There was no statistically significant association between trainee salary for Psychiatric Mental Health and clinical profession (χ2(6) = 6.371, p = 0.408), region (χ2(8) = 8.107, p = 0.494) sponsoring organization of the APP postgraduate training program (χ2(8) = 7.086, p = 0.614), and postgraduate APP trainee cohort (χ2(2) = 0.438, p = 1.000).

**Table 6:** Trainee salary for Psychiatric Mental Health by demographics The data has been represented as N: sample size, Df: degrees of freedom, χ2: chi-squared, P: p-value, p-value is considered significant (p<0.05, p<0.001). APP: advanced practice provider

Category	Psychiatric Mental Health (N = 26)					
	Below $75000	$76000-$85000	Over $86000	χ^2^	df	p
Clinical profession						
Nurse Practitioner (NP), N = 22	4 (18.2)	10 (45.5)	8 (36.4)	6.371	6	0.408
Physician Assistant/Associate (PA), N = 1	1 (100)	0	0	-	-	-
Physician, N = 1	0	0	1 (100)	-	-	-
N/A: Non-clinical, N = 2	0	1 (50.0)	1 (50.0)	-	-	-
Region						
Midwest, N = 6	2 (33.3)	3 (50.0)	1 (16.7)	8.107	8	0.494
West, N = 5	0	3 (60.0)	2 (40.0)	-	-	-
Southeast, N = 3	0	2 (66.7)	1 (33.3)	-	-	-
Southwest, N = 4	2 (50.0)	0	2 (50.0)	-	-	-
Northeast, N = 8	1 (12.5)	3 (37.5)	4 (50.0)	-	-	-
Sponsoring organization of the APP postgraduate training program						
Academic Medical Center, N = 4	1 (25.0)	2 (50.0)	1 (25.0)	7.086	8	0.614
Federally Qualified Health Center (FQHC), N = 7	1 (14.3)	2 (28.6)	4 (57.1)	-	-	-
U.S. Department of Veterans Affairs, N = 11	2 (18.2)	6 (54.5)	3 (27.3)	-	-	-
Hospital/Multi-Hospital System, N = 2	1 (50.0)	1 (50.0)	0	-	-	-
Other, N = 2	0	0	2 (100)	-	-	-
Composition of the postgraduate APP trainee cohort						
NP only, N = 23	4 (17.4)	10 (43.5)	9 (39.1)	0.438	2	1.000
PA only	0	0	0	-	-	-
Joint NP/PA, N = 3	1 (33.3)	1 (33.3)	1 (33.3)	-	-	-

Table 7 shows the results of Kendall’s tau-b correlation coefficients for trainee salary and number of postgraduate APP trainees. There was a statistically significantly positive relationship between trainee salary and the number of postgraduate APP trainees in Psychiatric Mental Health (τb = 0.451, p = 0.006). However, there was no statistically significant positive relationship between trainee salary and the number of postgraduate APP trainees in Primary Care (τb = 0.165, p = 0.113).

**Table 7:** Kendall’s tau-b for trainee salary and number of postgraduate APP trainees N: sample size: τb: Kendall’s tau-b: p: p-value. * indicates statistical significance at the 0.05 level.

Specialty	N	τ_b_	p
Primary Care	61	0.165	0.113
Psychiatric Mental Health	26	0.451	0.006*

## Discussion

To the best of our knowledge, this is the first cross-sectional survey that exclusively examines and summarizes trainee salary data from various APP postgraduate specialty training programs. In addition, we investigated whether there was an association between trainee salary by specialty track and the other four variables (i.e., program type, geographical location, clinical setting, and number of postgraduate APP trainees). Data from 37 specialty tracks were summarized but only two specialty tracks (Psychiatric Mental Health and Primary Care) were included in the association analysis due to sample size; it is important to note that slightly less than 30% of respondents were associated with FQHCs or lookalikes. These are safety net providers that deliver prevention and provide healthcare services through primary care clinics to underserved communities [19].

In terms of the number of trainees, our results indicate that the minimum number of postgraduate APP trainees per specialty track was one and the maximum number of postgraduate APP trainees per specialty track was 11. Regarding trainee salaries, most postgraduate programs in our study offer an annual salary between $65,000 and 85,000, which is lower than a career full-time NP or PA. In addition, there was a statistically significant association between trainee salary for Primary Care and clinical profession (χ2(6) = 13.993, p = 0.022). Respondents who were NPs (52.1%) reported that their trainees had an annual salary between $76000 and $86000. The majority of PA respondents (57.1%) reported that their trainees had an annual salary below $75000. Respondents who were non-clinical professionals (50.0%) reported that their trainees had an annual salary of over $86000. The single physician respondent also reported that their trainees had an annual salary of over $86000. It appears that respondents who were PAs were more likely to report lower trainee salaries than respondents who were NPs and non-clinical professionals.

Additionally, there was a statistically significant association between trainee salary for Primary Care and postgraduate APP trainee cohort (χ2(2) = 7.802, p = 0.016). Respondents having NP-only cohorts (54.5%) reported that their trainees had an annual salary within the range of $76000 and $85000. Respondents having joint NP/PA cohorts (55.6%) reported that their trainees had an annual salary of over $86000. It appears that participants having joint NP/PA cohorts were more likely to report higher trainee salaries than respondents from NP-only cohorts. No data for participants with PA-only cohorts were available for Primary Care. Not surprising, as PA residency/fellowship postgraduate programs are more concentrated in medical and surgical specialties/subspecialties than primary care.

Lastly, the study results indicated that for Psychiatric Mental Health, organizations with more postgraduate APP trainees tended to offer higher trainee salaries. There was no association between trainee salaries and geographic region in this study. Understanding how trainee salaries are determined and whether they are aligned with regional costs of living would be helpful to APP postgraduate programs [20,21]. Unfortunately, without more information, our observations regarding the data sample are limited.

Therefore, these rare findings underscore the critical need for a more detailed analysis of trainee salaries including fringe benefits across various specialty training programs. Areas of future research should include whether postgraduate APP trainee salaries are determined by any of the following factors: adjusted cost of living, supply and demand, projected minimum level of productivity, collective bargaining agreement, specialty or subspecialty type, and/or through a compensation survey. Another area of investigation is whether postgraduate APP trainees in rural underserved areas earn more or less than those in major metropolitan areas. Whether APP postgraduate trainee salaries vary by gender is another area of interest. Finally, it is important to understand whether APP trainee financial compensation influences the choice of a specific specialty or subspecialty. Prior research has found that medical residents are attracted to specialties that offer annual vacations, higher earnings, shorter residency programs, and predictable work schedules but a similar study has not been conducted on postgraduate APP trainees [22].

Limitations

Not dissimilar to other studies, our findings were limited solely to trainee salaries from a small sample size, and therefore, our results may not be generalizable [23]. Also, the low response rate of 45% may have led to non-response bias. In addition, PA postgraduate programs were underrepresented in the study and their perspective may have influenced the study results. Another limitation is that some respondents disclosed trainee salaries and the number of trainees for some but not all specialty tracks. This led to some incomplete responses. Further, multi-specialty track program trainee salaries may vary depending on the specialty track, a large study that examines multi-specialty track trainee salaries is warranted. Lastly, we did not define primary care in the survey which may have caused confusion due to overlap with other similar specialty tracks. Given these limitations, the study's findings should be interpreted with caution.

## Conclusions

In conclusion, this novel study sheds some light on postgraduate APP annual salaries including calculated associations between Primary Care and Psychiatric Mental Health. However, more research is warranted to understand the role of other variables on postgraduate trainee salaries and benefit compensation to better inform program directors and policymakers. Lastly, an attempt should be made to investigate postgraduate trainees’ perceptions regarding their salaries and fringe benefits.

## Appendices

**Table 8:** Study questionnaire Analysis and summary of 8 out of 29 survey questions (29.6%).

Numbers	Study Questionnaire
1	Please indicate your clinical profession.
2	In what state is your organization’s APP postgraduate training program located?
3	Please describe the sponsoring organization of your APP Postgraduate Training Program.
4	If the setting of your organization's APP Postgraduate Training Program was not listed in the previous question, please indicate the setting below.
5	What is the composition of your Postgraduate APP Trainee Cohort?
6	How many postgraduate APP trainees are in each program track cohort?
7	If the cohort size is 11+, please indicate the program track cohort size and specialty.
8	Please indicate the specialty track of your postgraduate APP trainees and salary range using the options below (59 specialty tracks listed).
